# Probability Density Function Models for Float Glass under Mechanical Loading with Varying Parameters

**DOI:** 10.3390/ma16052067

**Published:** 2023-03-02

**Authors:** Evelien Symoens, Ruben Van Coile, Balša Jovanović, Jan Belis

**Affiliations:** Magnel-Vandepitte Laboratory, Department of Structural Engineering and Building Materials, Ghent University, Technologiepark-Zwijnaarde 60, 9052 Gent, Belgium

**Keywords:** structural glass, crack prediction, strength prediction, numerical modelling, parameter study

## Abstract

Glass as a construction material has become indispensable and is still on the rise in the building industry. However, there is still a need for numerical models that can predict the strength of structural glass in different configurations. The complexity lies in the failure of glass elements largely driven by pre-existing microscopic surface flaws. These flaws are present over the entire glass surface, and the properties of each flaw vary. Therefore, the fracture strength of glass is described by a probability function and will depend on the size of the panels, the loading conditions and the flaw size distribution. This paper extends the strength prediction model of Osnes et al. with the model selection by the Akaike information criterion. This allows us to determine the most appropriate probability density function describing the glass panel strength. The analyses indicate that the most appropriate model is mainly affected by the number of flaws subjected to the maximum tensile stresses. When many flaws are loaded, the strength is better described by a normal or Weibull distribution. When few flaws are loaded, the distribution tends more towards a Gumbel distribution. A parameter study is performed to examine the most important and influencing parameters in the strength prediction model.

## 1. Introduction

In the building industry, soda–lime–silica glass has been mostly used as infill panels, but now more and more commonly as a structural component. As such, one of its most significant challenges is the brittle behavior, resulting in an instantaneous fracture. This challenge can be partly tackled by lamination or combining the glass with, e.g., steel reinforcement [1,2,3,4,5]. However, predicting the behavior of a structural glass panel and its corresponding failure strength is not easy because of the presence and randomness of pre-existing surface flaws on the glass surface, known as ‘Griffith flaws’ [6,7,8,9]. These flaws initiate the failure of the glass once its maximum tensile strength is exceeded. They are created during production, transportation, cutting, finishing, etc., and their properties vary from flaw to flaw. Consequently, the fracture strength of the glass panels may be described by a probability function, which will depend on the size of the glass panels and the loading conditions.

Due to this complexity, glass plates are frequently modeled with a deterministic fracture strength, making the calculation easier. However, this approach does not properly predict the failure strength of glass and does not allow for an explicit evaluation of the safety level (e.g., to allow for a reliability-based design format). Therefore, models need to predict the probabilistic failure strength of the glass in all kinds of configurations.

Some models are already available in the literature, going from very advanced models to more simplified models [10,11,12]. One of the latter is a strength prediction model of annealed glass proposed by Yankelevsky [12] and later adopted by Osnes [13]. The analysis obtains the strength of glass panels as a result and not as an assumption, which is an advantage compared to advanced models. This model predicts the failure of glass elements by using a crack initiation criterion. The crack initiates when a stress intensity factor exceeds a fracture toughness, resulting in the total failure of the glass plate. An advantage of the model is its ability to easily model different setups.

The current work extends this approach by adding a probability density model selection to the simulations. The Akaike information criterion (AIC) is chosen since model estimation and selection are simultaneously accomplished [14,15]. This allows us to find the most appropriate distribution fit for all the generated data. In addition, a parametric study has been carried out with this model to identify which parameters have a key influence on the probabilistic fracture strength.

## 2. Materials and Methods

The following section describes a numerical model called the strength prediction model (SPM), where failure of glass is predicted using a crack initiation criterion. First, glass, in general, is briefly discussed, followed by an explanation of the general procedure of the SPM. Next, the different parameters of the SPM are discussed with their assumed values. Subsequently, a case study will be presented, which will be used to demonstrate the different steps in the model. Finally, the outcome of the numerical SPM is validated with the experimental data of the presented case study, followed by a convergence study of that outcome.

### 2.1. Glass and Properties

The most commonly used glass in construction is soda–lime–silica (SLS) glass. Some of its characteristics are given in Table 1.

Every glass surface contains imperfections that are not visible to the naked eye. These imperfections are called surface flaws and are created during manufacturing, post-processing, packaging, transport, and service life. Flaws on glass surfaces are typically assumed to have an elliptical shape [16] and cause glass panes to fail when subjected to tension perpendicular to the flaw orientation. Due to this tension, the flaws will open up and initiate a crack in the glass.

**Table 1:** Some thermal and mechanical characteristics of annealed soda–lime–silica glass [17,18,19].

Characteristic	Symbol and Unit	Value
Density	ρ [kg/m³]	2500 [18]
Glass transition temperature	T_g_ [°C]	540–575 [17,19]
Coefficient of thermal expansion	α_T_ [10^−6^ °C^−1^]	8.9–9 [17,18]
	(T < T_g_)	
Young’s modulus	E [GPa]	70–73 [17,18]
Poisson’s ratio	ν [−]	0.23 [17]
Thermal conductivity	λ [W/mK]	1 [18]
Nominal tensile resistance	σ_t_ [MPa]	45 [18]

### 2.2. Strength Prediction Model

A crack in a glass pane is initiated when the Mode I stress intensity factor $K_{I}$ of a flaw reaches the fracture toughness $K_{Ic}$ [16]. This crack initiation criterion is presented in Equation (1). The stress intensity factor is calculated with Equation (2), where Y stands for the geometry factor, σ is the stress perpendicular to the flaw orientation and a represents the flaw depth [16]. The geometry factor Y is a dimensionless factor that is dependent on the geometry of the flaw. Further background on the calculation of Y and the other parameters in this crack initiation criterion is given in Section 2.2.2.

(1) $$K_{I}=K_{Ic}$$

(2) $$K_{I}=Y\sigma \sqrt{\pi a}$$

#### 2.2.1. Procedure

Glass panes will only fail if flaws on their surface are subjected to tension perpendicular to the flaw orientation. To numerically simulate the crack initiation, surface flaws must first be distributed over the glass surfaces, each with a flaw orientation $\alpha_{i}$ and a flaw depth $a_{i}$. Once these flaw orientations and depths are generated, they are distributed over the glass surfaces. Next, a mechanical load is applied, which induces stresses. From these stresses, the normal stress σ for each flaw (i.e., the stresses perpendicular to the flaw orientation) can be calculated by Equation (3). The stresses $\sigma_{x}$, $\sigma_{y}$ and $\tau_{xy}$ can be calculated using a finite element (FE) program such as Abaqus [20] or calculated analytically for simple configurations.

(3) $$\sigma =\frac{\sigma_{x}+\sigma_{y}}{2}+\frac{\sigma_{x}-\sigma_{y}}{2}\cos\left(2\alpha_{i}\right)+\tau_{xy}\sin\left(2\alpha_{i}\right)$$

**Figure 1:**
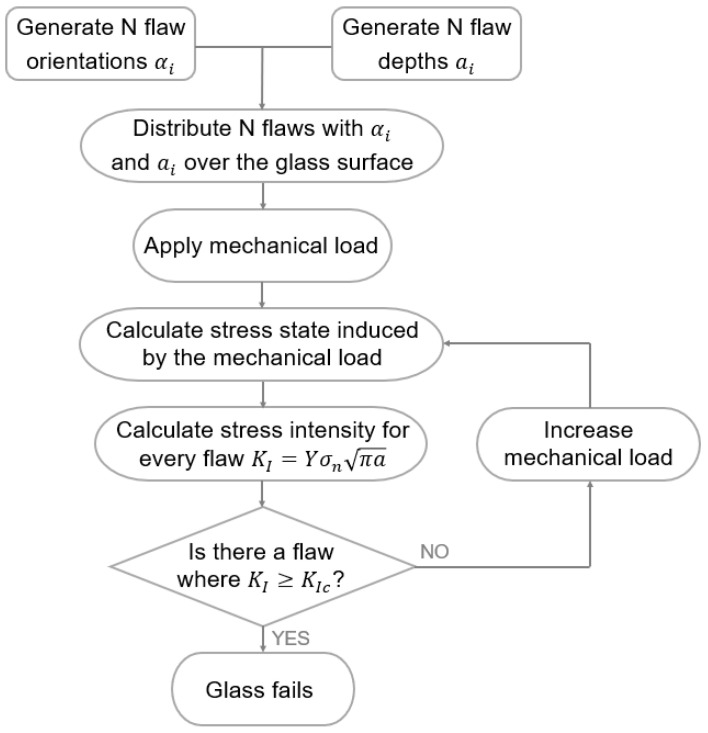
Flowchart for the evaluation of the glass strength using the strength prediction model.

Once the normal stresses are calculated, Equation (2) can be evaluated at a specific flaw location considering the corresponding flaw depth $a_{i}$ and a geometry factor Y. This results in a stress intensity factor $K_{I}$ for each flaw. Subsequently, the crack initiation criterion of Equation (1) is evaluated for each flaw. If the corresponding stress intensity factor $K_{I}$ exceeds the fracture toughness $K_{Ic}$, the fracture is initiated in that particular flaw, resulting in fracture of the total glass panel. On the other hand, if the crack initiation criterion is not met, the mechanical load is increased, resulting in increased stresses σ and increased stress intensity factors $K_{I}$ to eventually check again if one of these stress intensity factors $K_{I}$ exceeds the fracture toughness $K_{Ic}$. This procedure is repeated until the glass eventually fractures, resulting in an evaluation of the maximum load for the glass pane. A flowchart of this procedure is presented in Figure 1.

#### 2.2.2. Parameters

The SPM relies on the dimensions of the setup and six other parameters: the fracture toughness, the flaw density, the flaw orientation, the flaw depth, the maximum flaw depth, and the flaw shape. The fracture toughness, at inert conditions, $K_{Ic}$ is fixed for an elliptical flaw as $0.75\;\mathrm{MPa}\sqrt{m}$ [18,21].

To quantify the number of flaws simulated on the glass surfaces, the flaw density parameter $\rho_{flaw}$ is used, representing the number of flaws present in one square centimeter. The literature shows that two flaws for each square centimeter are a good assumption [13,22].

With this information, the next two parameters can be calculated: the flaw orientation $\alpha_{i}$ and the flaw depth $a_{i}$ for each flaw. The former represents a random value uniformly distributed between zero and π, and the latter represents a random value between zero and the maximum flaw depth $a_{max}$. 

It may be assumed that the flaws distribution function is of an exponential shape which can be expressed with the following Equation (4) [12,23]:

(4) $$\frac{N_{i}}{N_{0}}=exp\left(-\frac{a_{i}}{\eta }\right)$$

where $N_{0}$ represents the number of surface flaws present on a jumbo glass plate (3210 mm by 6000 mm), $N_{i}$ the number of flaws that are larger or equal to the maximum flaw depth $a_{max}$, $a_{i}$ the size of a certain flaw on the glass plate, and η the distribution parameter.

Suppose $a_{i}$ = $a_{max}$; it means $N_{i}$ will be equal to 1. Substituting this condition in Equation (4), one obtains the distribution parameter η equal to $a_{max}$/ln($N_{0}$) [12]. With this information, Equation (4) can be rewritten to determine the random depth value $a_{i}$ for each flaw as the following Equation (5):

(5) $$a_{i}=a_{max}\left(1-\frac{\ln\left(N_{i}\right)}{\ln\left(N_{0}\right)}\right)$$

where $N_{i}$ is selected with the expression $N_{i}=R\left(N_{0}-1\right)+1$ in which R is a random value uniformly distributed between zero and one. The maximum flaw depth parameter $a_{max}$ is considered equal to 0.1 mm [13].

**Figure 2:**
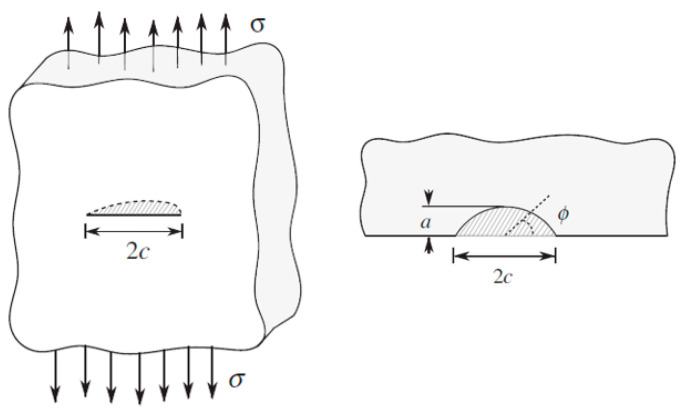
Representation of an elliptical surface flaw [13].

Next, information about the geometry of the flaw is needed to calculate the geometry factor Y. However, these flaws can have various shapes, so some assumptions must be made until more information is available. Therefore, it is chosen to model the flaws on the glass surface with an elliptical shape [12], as depicted in Figure 2. The model assumes the flaws are located in the center of each square centimeter of the surfaces. Levengood [23] stated that for Griffith flaws, one-half of the crack length c was observed to be of the same order of magnitude as the crack depth a. Based on this, the parameter of flaw depth on half length of the flaw a/c for elliptical flaws is considered 1 and is called the flaw shape.

The geometry factor Y of the elliptical flaws considered here is calculated using Equation (6), where $\lambda_{s}$ is the surface correction factor, $f\left(\emptyset \right)$ is the angular function and Q is the flaw shape parameter [6,13,16].

(6) $$Y=\frac{\lambda_{s}f\left(\emptyset \right)}{\sqrt{Q}}$$

All the parameters in Equation (6) are calculated using the empirical expressions (7) to (9), with ∅ equal to zero, which stands for the angle of the point on the elliptical flaw at which the stress intensity factor is at its maximum. Taking this into account, the final geometry factor Y for elliptical flaws, calculated with Equation (6), is equal to 0.729 [6,13,16].

(7) $$\lambda_{s}=\left[1.13-0.09\left(\frac{a}{c}\right)\right]\left[1+0.1{\left(1-sin\emptyset \right)}^{2}\right]$$

(8) $$Q=1+1.464{\left(\frac{a}{c}\right)}^{1.65}$$

(9) $$f\left(\emptyset \right)={\left[sin^{2}\left(\emptyset \right)+{\left(\frac{a}{c}\right)}^{2}cos2\left(\emptyset \right)\right]}^{\frac{1}{4}}$$

#### 2.2.3. Case Study

To demonstrate the different steps in the SPM, a case study performed by Osnes et al. [13] will be used. The experimental data of this case study will be used to validate the outcome of the SPM at the end of this section.

The case study is a four-point bending test performed on glass specimens of 300 mm long, 60 mm wide and 4 mm thick, where the support span $L_{s}$ is equal to 280 mm and the loading span $L_{l}$ is equal to half of the support span, i.e., 140 mm. The dimensions of the setup and the specimens are based on the ASTM standard C1161-13 [24], originally intended for testing advanced ceramics. 

#### 2.2.4. SPM Applied on Case Study

**Figure 3:**
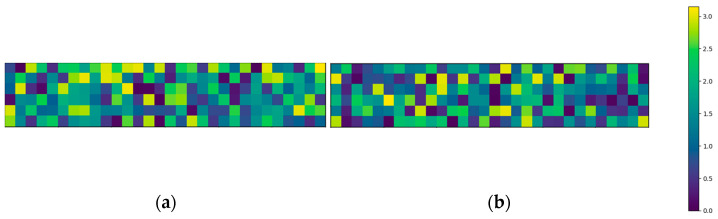
Flaw orientation distribution for (**a**) top and (**b**) bottom surfaces (with a flaw density of 1 flaw/cm^2^ for visualization).

**Figure 4:**
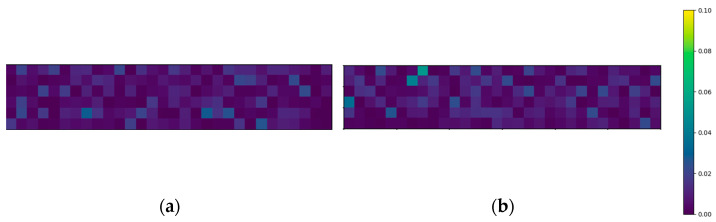
Flaw depth distribution for (**a**) top and (**b**) bottom surfaces (with a flaw density of 1 flaw/cm^2^ for visualization).

The above-mentioned four-point bending setup is modeled in Python. The area of the glass panel combined with the flaw density $\rho_{flaw}$ results in the total amount of flaws present on one glass surface. With this information, the flaws can be randomly distributed over both the top and bottom surfaces. Each flaw can be assigned a random flaw orientation $\alpha_{i}$ and a random flaw depth $a_{i}$ as discussed in Section 2.2.2. To demonstrate this concept, Figure 3 and Figure 4 visualize the distribution of the flaw orientations and depths, respectively. To display this clearly, these visualizations are divided into elements of one square centimeter and exceptionally adopt the simplification of displaying one color for each square centimeter, i.e., a flaw density of one flaw/cm^2^.

Next, the mechanical load is applied which will induce stresses. As mentioned, these stresses can be calculated using an FE program such as Abaqus or calculated analytically. For visualizing purposes, Abaqus is used here to calculate the stresses.

**Figure 5:**
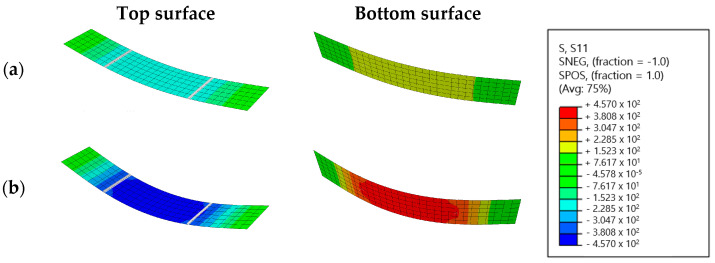
The $\sigma_{x}$ stress states of the top and bottom surfaces for a total load of (**a**) 1000 N and (**b**) 1800 N.

Both the four-point bending setup and the increased mechanical loading are numerically simulated. This simulation’s output are the stress states induced by the mechanical loading at evenly spaced loading intervals and for every location on the glass pane. Stress states consist of the in-plane normal stresses in the x and y direction, respectively, called $\sigma_{x}$ and $\sigma_{y}$, and the in-plane shear stress $\tau_{xy}$. In Figure 5, the $\sigma_{x}$ stress states of the bottom and top surfaces are visualized for a total load of 1000 N and 1800 N.

**Figure 6:**
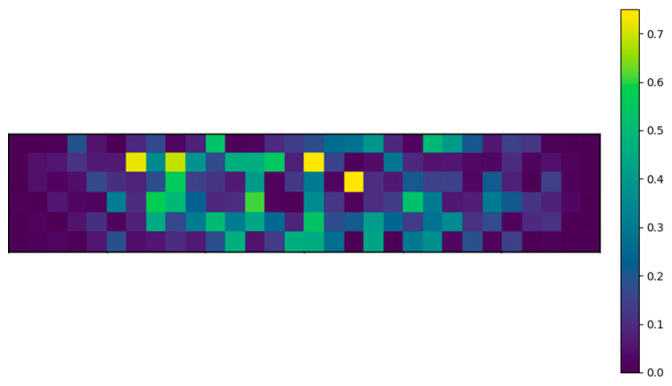
Stress intensity factor for bottom surface (with a flaw density of 1 flaw/cm^2^ for visualization).

**Figure 7:**
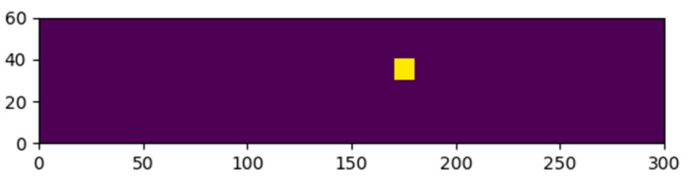
Fracture location (yellow) for bottom surface (axis dimension in mm).

These $\sigma_{x}$, $\sigma_{y}$ and $\tau_{xy}$ stress states are used to calculate the normal stress of each flaw for any load on the structure, as demonstrated in Equation (3). Combining this stress evolution with the corresponding flaw depths $a_{i}$ and the geometry factor Y as calculated in Equation (6), the evolution of the stress intensity factor $K_{I}$ for every flaw can be calculated and visualized, as depicted in Figure 6. The evolution of the stress intensity factor $K_{I}$ will show the location where a first flaw reaches a stress intensity factor equal to the fracture toughness of $0.75\;\mathrm{MPa}\sqrt{m}$. Once this failing flaw is found, one knows the fracture load and the fracture location for this particular glass pane in a four-point bending setup. Figure 7 visualizes the fracture location for the random glass pane shown in the former figures. In this example, the fracture originated at the bottom surface location with x and y coordinates equal to 175 mm and 35 mm, respectively.

#### 2.2.5. Probability Density Distribution

**Figure 8:**
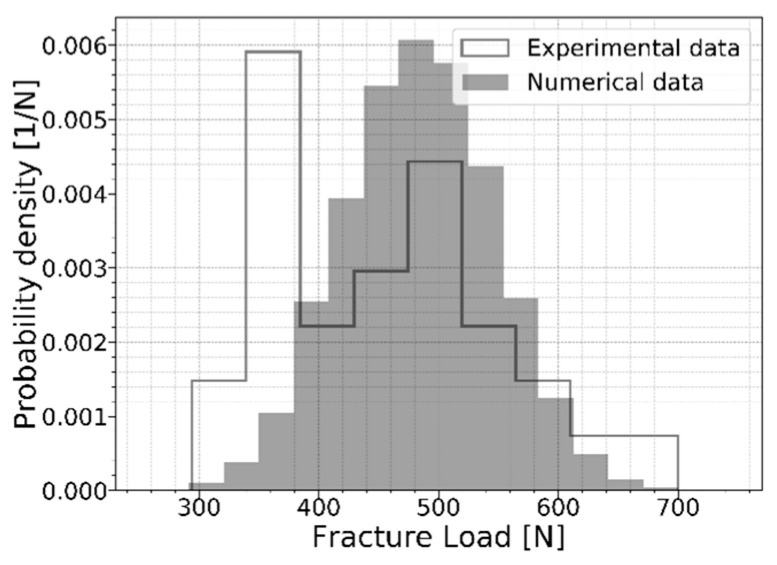
The normalized histogram of the fracture load.

**Figure 9:**
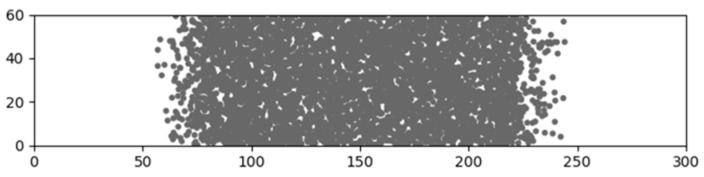
The fracture locations of 5000 numerical repetitions.

Once the critical flaw is found, the fracture load and location are known. The crack calculation for a random glass pane can be repeated multiple times to obtain a histogram of the outcomes. Figure 8 presents the normalized histogram of this fracture load for 5000 repetitions, with a flaw density of two flaws for each square centimeter. The x-axis represents the fracture load, and the y-axis represents the observed probability density. The latter is a normalized value calculated by Equation (10), where $w_{i}$ is the bins’ width in the distribution, $c_{i}$ represents the number of observations in the respective bin, and N is the total number of tests. The corresponding fracture locations for 5000 repetitions are illustrated in Figure 9.

(10) $$\frac{c_{i}}{N\cdot w_{i}}$$

To validate this outcome, the numerical results are compared to experimental results in Figure 8. Osnes et al. [13] performed 30 tests in the presented four-point bending setup and recorded the applied load at failure for each test. Comparing the numerical and experimental results, it can be concluded that the numerical model described in this paper predicts the strength of glass panes properly. However, the experimental results indicate a larger frequency of small fracture loads and overall show a larger variability. At this point, there is no clear justification for this. Furthermore, Osnes et al. obtained a similar discrepancy with the experimental data. This is thus a point requiring further research. With this information, the description of the strength prediction model is considered completed.

**Figure 10:**
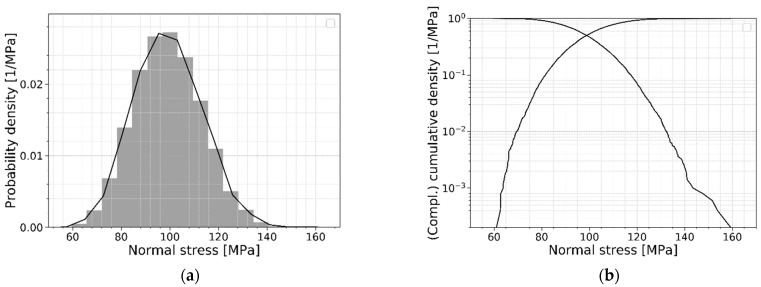
(**a**) Probability density and (**b**) (compl.) cumulative density of the normal stress.

Next, another parameter can be derived from the numerical model: the stress perpendicular to the flaw orientation of the failing flaw. This normal stress is an important parameter since it indicates the tensile capacity of the subjected flaws. The observed probability density and the cumulative density of this normal stress are shown in Figure 10. This normal stress distribution will be used to perform a convergence study and several parameter studies described later in this paper.

#### 2.2.6. Convergence Study

**Figure 11:**
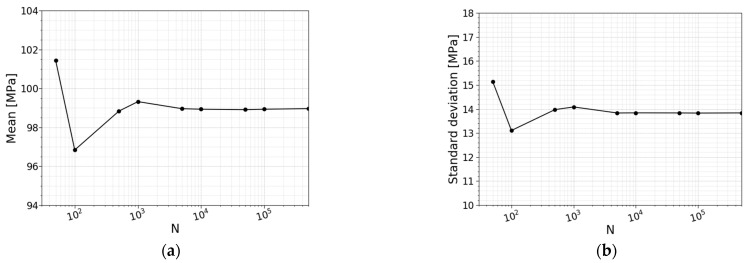
Convergence of (**a**) the mean value and (**b**) the standard deviation.

To ensure that the strength prediction model’s output is not affected by the number of simulations, a convergence study is performed. In this study, the output of the numerical model will converge to a repeatable solution when increasing the number of simulations. As mentioned, the normal stress distribution will be used to perform this convergence study. Table 2 lists the models used for this convergence study. For each model, the number of simulations is mentioned together with the calculated mean value of the normal stress distribution, the standard deviation and the coefficient of variation (COV). Figure 11 visualizes the convergence of the mean value and the standard deviation. From Table 2 and Figure 11, from 5000 simulations onwards, a reliable result in the overall shape of the distribution and its corresponding mean value and standard deviation can be obtained.

**Figure 12:**
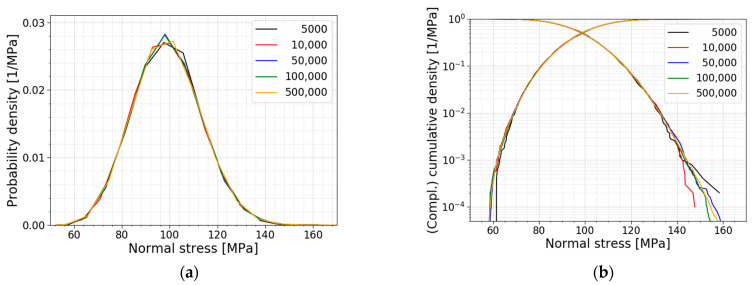
(**a**) PDF and (**b**) CDF of the convergence study.

Figure 12 presents the PDFs and the CDFs of the above-mentioned models with the number of simulations starting from 5000 simulations. This figure clearly illustrates that even more simulations are needed to obtain a reliable result at the lower quantiles. To calculate the required number of simulations for a specific quantile, Equation (11) is adopted, and the corresponding results are displayed in Table 3. In Equation (11), N represents the number of simulations needed, $P_{q}$ is the quantile of interest and $COV_{q}$ is the target coefficient of variation for the quantile probability (set equal to 0.14 here to obtain a comparable number of simulations as in Figure 12). Such a quantile evaluation is relevant when the theoretical distribution describing the glass strength is unknown. However, as soon as a theoretical distribution is assigned, quantiles can be easily evaluated as soon as the parameters of the distribution (e.g., mean and standard deviation) are known.

(11) $$N=\frac{1-P_{q}}{P_{q}\cdot COV_{q}^{2}}$$

### 2.3. Model Selection and Distribution Fitting—AIC Calculation

To generalize the results, a distribution must be plotted on the outcome of the SPM, i.e., the probability density and the cumulative density of the normal stresses. Osnes [13] plotted a normal distribution of the data outcome of the SPM. It is observed that this normal distribution fits well with the data of a four-point bending setup with a length of 300 mm and a width of 60 mm. However, the data of a four-point bending setup with a length of 100 mm and a width of 20 mm differ from the normal distribution fit. This highlights the importance of investigating the right theoretical model and its possible dependence on the simulated case.

To select the right model, several criteria are available such as the Bayesian information criterion (BIC), the Akaike information criterion (AIC), and the minimum description length (MDL) [15]. In this paper, the Akaike information criterion (AIC) is chosen since model estimation and model selection are simultaneously accomplished [15]. The criterion was introduced in 1971 by Hirotugu Akaike, and the criterion is defined by an AIC value calculated according to Equation (12) for each model [25]. In Equation (12), k is the number of estimated parameters in the model and $\hat{L}$ is the maximum value of the likelihood function for the model. Once the AIC values of the competing models are calculated, the most appropriate model is that with the lowest AIC value.

(12) $$AIC=2\cdot k-2\cdot ln\left(\hat{L}\right)$$

In this paper, two-parameter distributions will be evaluated, which makes the value k equal to 2. The competing models are a normal distribution, a lognormal distribution, a gamma distribution, a Gumbel distribution and a two-parameter Weibull distribution.

## 3. Results and Discussion

**Figure 13:**
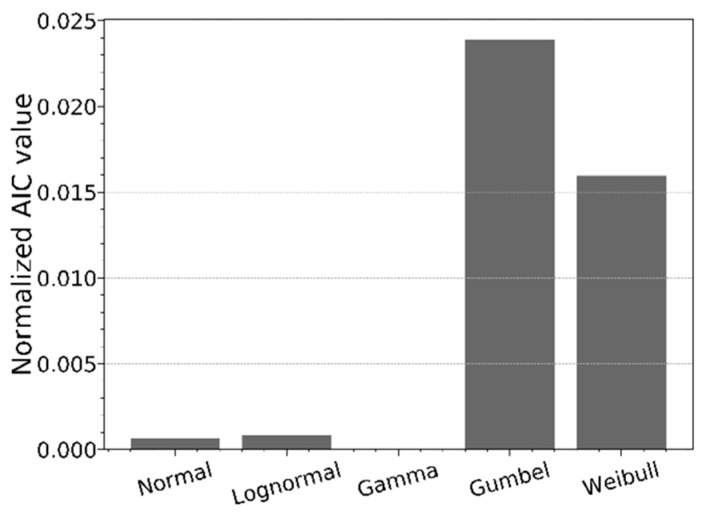
AIC values of the competing models.

**Figure 14:**
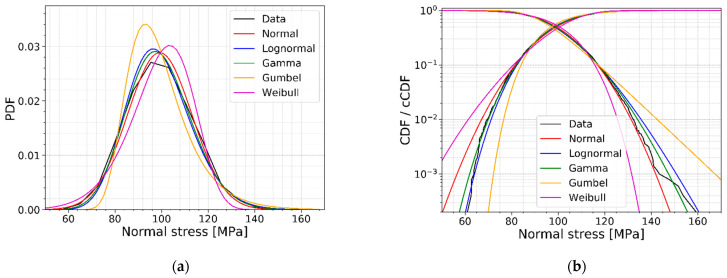
(**a**) PDFs and (**b**) CDFs of the competing models with the outcome data.

The information in Section 2.3 allows us to select the most appropriate model to represent the data of the discussed case study in Section 2.2.3. Figure 13 indicates the normalized AIC values for the competing distributions, as discussed in Section 2.3, fitted on the data of Figure 10. From Figure 13, it can be concluded that the gamma distribution has the lowest AIC value, resulting in the most appropriate model to represent the data of Figure 10. Figure 14 illustrates the data of Figure 10 together with the five different models. It is clearly visible that the gamma and lognormal distributions are the best fit, especially for the lower quantiles. The normal distribution underestimates the strength (normal stress at fracture) for the lower quantiles.

With the validated strength prediction model and the above-mentioned information regarding the convergence and distribution fitting, different parameter studies are performed to give an idea of the most sensitive parameters in the model. The parameters that will be examined below are, in order of appearance, the scale factor, the ratio width/loading span, the ratio loading span/support span, the flaw density, the flaw shape, the maximum flaw depth and the fracture toughness.

For every parameter, a table will present the varying parameter value together with the corresponding mean value, standard deviation, and COV for the normal stress at failure and the best fit (i.e., the distribution with the lowest AIC value). The setup introduced in Section 2 serves as a reference. This reference is displayed in every table of the parameter studies by means of a table footer.

### 3.1. Scale Factor

**Figure 15:**
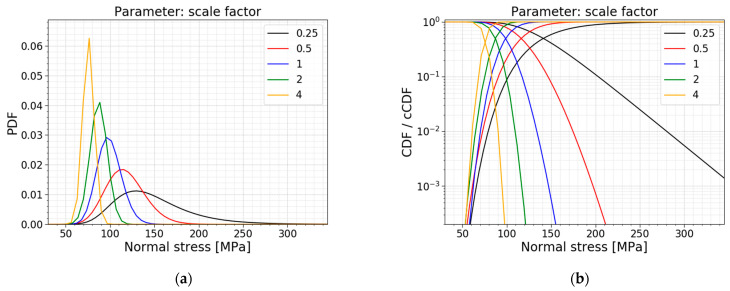
(**a**) PDF and (**b**) CDF for a varying scale factor.

**Figure 16:**
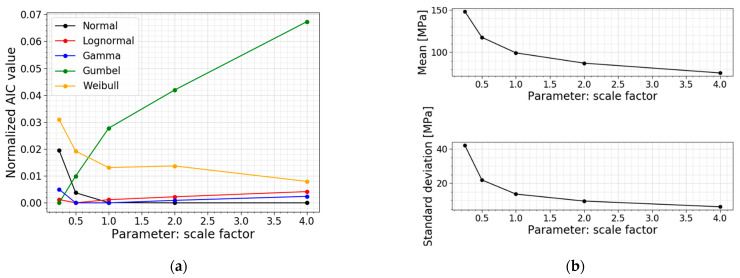
(**a**) AIC values and (**b**) the mean and st.dev. of the normal stress at failure for a varying scale factor.

The investigation is a study on the total scaling of the setup. The width and the support span are scaled with the same factor, while the loading span is kept constant as half of the support span. The obtained results are presented in Table 4, the corresponding PDFs and CDFs are visualized in Figure 15, and the trends of the normalized AIC values, the mean values and the standard deviations are presented in Figure 16.

Table 4 and Figure 16b conclude that the mean value, the standard deviation, and COV decrease when a larger setup is considered, and vice versa. The larger the setup, the more flaws are present and are subjected to large tensile stresses in the loading span. This ensures a larger probability for a deep flaw to be present, which causes the glass to fail at a lower fracture load, which goes hand in hand with lower normal stresses at the moment of failure, as was expected. This trend is also clearly visible in Figure 15; the higher the scaling factor, the more the distribution shifts to the left and becomes more narrow.

Another observation is the shift in the best distribution fit. From Table 4, it can be concluded that the data of the small setup (i.e., the setup with a scaling factor of 0.25) fits best to a Gumbel distribution. When looking at the data of the setups with a scaling factor of 0.5–1, the best fit changes to a gamma distribution, and for the data of the biggest setups (i.e., the setups with a scaling factor of 2–4), to a normal distribution. This trend is also noticeable in Figure 16a, where all the normalized AIC values of the different models are summarized. This figure highlights that the Gumbel fit gets worse when approaching larger setups. The Weibull fit is the other way around; this fit gets better when approaching larger setups. For the performed scale factors, the gamma and lognormal fits perform well overall.

### 3.2. Width/Loading Span

**Figure 17:**
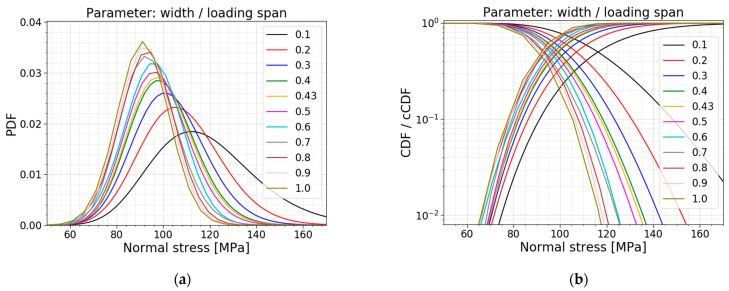
(**a**) PDF and (**b**) CDF for a varying ratio of the width to the loading span.

**Figure 18:**
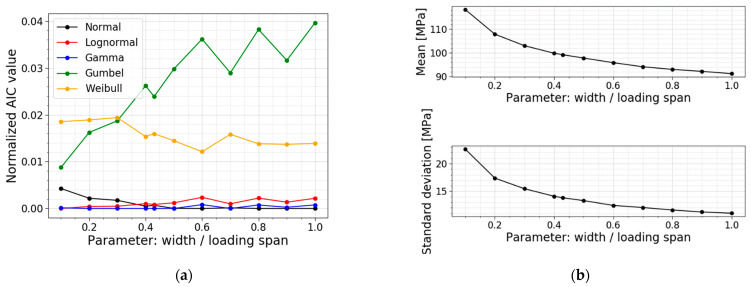
(**a**) AIC values and (**b**) the mean and st.dev. of the normal stress at failure for a varying ratio of the width to the loading span.

The next parameter is the ratio of the width to the loading span. The width and the loading span in the reference setup are equal to 60 mm and 140 mm, respectively, resulting in a ratio of 0.43. The other models are made while keeping the loading span constant (i.e., 140 mm) and varying the width parameter to influence the ratio. Table 5 displays the data of the different models, the corresponding PDFs and CDFs are visualized together in Figure 17, and the trends of the normalized AIC values, the mean values and the standard deviations are presented in Figure 18.

The table and Figure 18b display a decrease in the mean value, standard deviation and COV when increasing the ratio, and vice versa. For these simulations, a larger ratio is caused by a larger width, resulting in a larger region subjected to the maximum bending moment. This means that more flaws are present in the zone with the maximum stresses, increasing the probability of the glass failing at lower stresses. This trend is also clearly visible in Figure 17; the higher the ratio, the more the distribution shifts to the left and becomes more narrow. 

Observing the column with the best fit in Table 5, a shift for the best fit is noticeable. For the small ratios (i.e., slender samples), a lognormal distribution has the lowest AIC value. Ratio 0.2 to 0.4 corresponds best to a gamma distribution. In the range from 0.5 to 0.7, the AIC values for a normal fit and a gamma fit are very close to each other, so both fits can be used. The largest ratios, starting from 0.8, have the normal distribution as the lowest AIC value. Figure 18a illustrates the same trend. Additionally, the normalized AIC values visualize that the Gumbel fit becomes worse when approaching larger values for the ratio of the width to the loading span. Again, the gamma and lognormal fit perform well for all the parameter values considered.

### 3.3. Loading Span/Support Span

**Figure 19:**
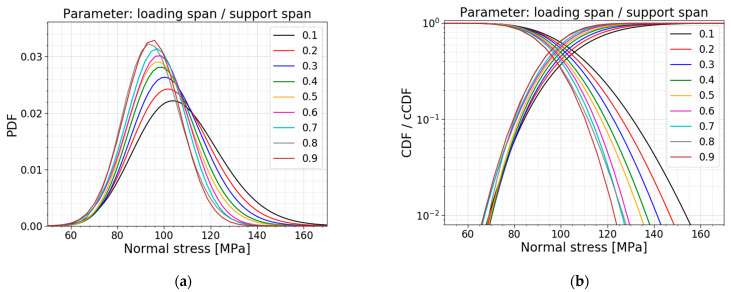
(**a**) PDF and (**b**) CDF for a varying ratio of the loading span to the support span.

**Figure 20:**
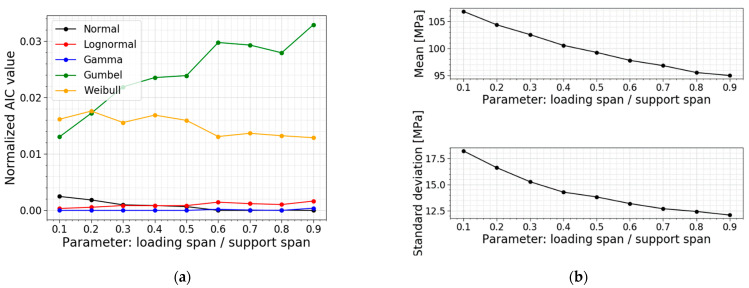
(**a**) AIC values and (**b**) the mean and st.dev. of the normal stress at failure for a varying ratio of the loading span to the support span.

Another parameter study is performed on the ratio of the loading span to the support span. The loading and support span in the reference setup is equal to 140 mm and 280 mm, resulting in a ratio of 0.5. The other models are made while keeping the support span constant (i.e., 280 mm) and varying the loading span parameter to influence the ratio. Table 6 displays the data for the models, all PDFs and all CDFs are visualized together in Figure 19, and the trends of the normalized AIC values, the mean values and the standard deviations are presented in Figure 20.

Table 6 and Figure 20b indicate a smaller mean value, standard deviation and COV for larger ratios, and vice versa. The larger the loading span, the larger the region subjected to the maximum stresses. This way, the number of flaws in this region is higher, increasing the probability of the glass failing at smaller stresses. Again, this trend is also clearly visible in Figure 19; the larger the ratio, the more the distribution shifts to the left and becomes more narrow.

A study of the column with the best fit tells us a shift in the best distribution fit. The data of the ratios below and equal to 0.5 all list the best fit with a gamma distribution. From the data of the ratios higher and equal to 0.6, both a normal distribution and a gamma distribution are good fits since the AIC values hardly differ. Figure 20a illustrates the same trend, with the extra information that the Gumbel distribution worsens when considering higher ratios. Overall, the gamma fit performs well.

### 3.4. Flaw Density

**Figure 21:**
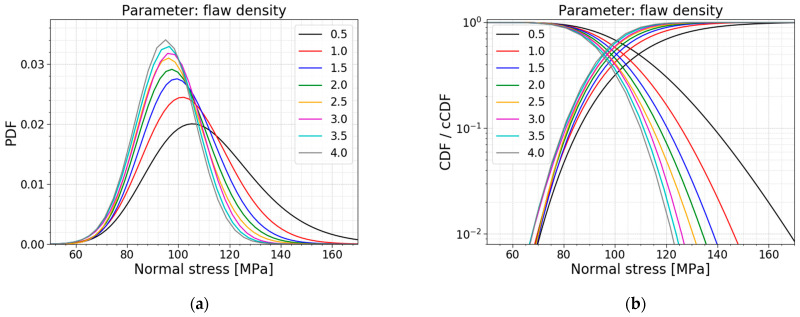
(**a**) PDF and (**b**) CDF for a varying flaw density.

**Figure 22:**
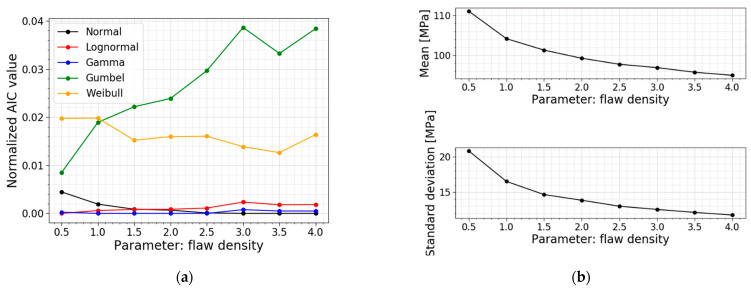
(**a**) AIC values and (**b**) the mean and st.dev. of the normal stress at failure for a varying flaw density.

In Section 2, the flaw density was presented as an integer. However, from the literature, it is clear that this parameter varies considerably, depending on the cutting and finishing processes used. Wereszczak et al., (2014) [22] investigated the flaw density for soda–lime–silica glass when scored and bent and cut with a water jet. The authors found that the flaw density can vary from 1.18 to 2.60 flaws/cm^2^. [22] This shows the importance of a parameter study on the flaw density. Table 7 displays the models used for this parameter study, all PDFs and all CDFs are visualized together in Figure 21, and Figure 22 presents the trends of the normalized AIC values, the mean values and the standard deviations.

Table 7 and Figure 22b prove that a larger flaw density causes a smaller mean value, standard deviation and COV for the normal stress distribution. A larger flaw density resulted in more flaws on the glass surface and subjected to the tensile stresses resulting from the bending moments. This ensures that there is a larger probability for a deep flaw to be present, which causes the glass to fail at a lower fracture load, which goes hand in hand with lower normal stresses at the moment of failure. Figure 21 visualizes the smaller values and a more narrow distribution for larger flaw densities. 

Another observation is again a switch in the best distribution fit. The data for a setup with a flaw density equal to 0.5 flaws/cm^2^ has the best fit with a lognormal distribution. For flaw densities ranging from 1.0 to 2.5 flaw/cm^2^, the best fit changes to a gamma distribution. Furthermore, flaw densities higher than and equal to 3.0 flaws/cm^2^ have a best fit with a normal distribution. The AIC values in Figure 22a also illustrate the Gumbel fit worsening when considering larger flaw densities.

### 3.5. Flaw Shape

**Figure 23:**
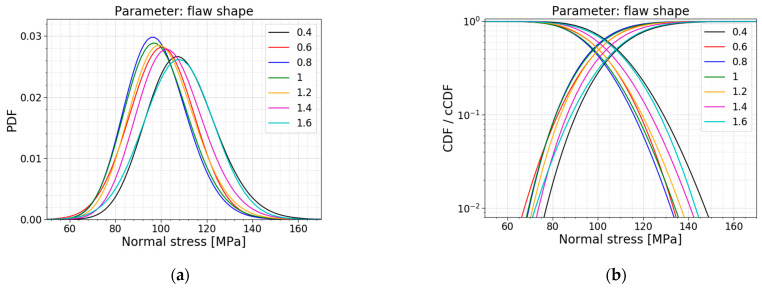
(**a**) PDF and (**b**) CDF for a varying flaw shape.

**Figure 24:**
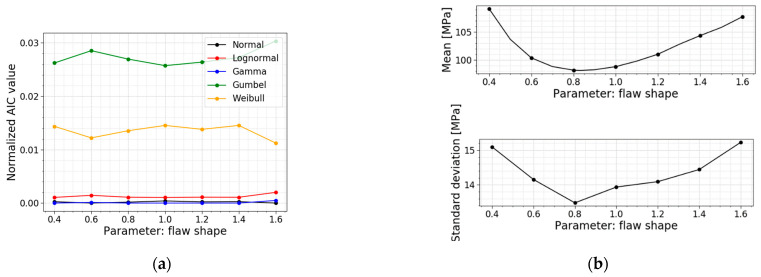
(**a**) AIC values and (**b**) the mean and st.dev. of the normal stress at failure for a varying flaw shape.

In the Section above, the flaw shape a/c is taken equal to 1 based on the work of Levengood [23]. Consulting Equations (6)–(9), as presented in Section 2.2.2, this assumption leads to a geometry factor of 0.729. However, this is an assumption where all flaws are idealized as elliptic. In reality, much more flaws will be irregularly shaped [12]. Therefore, in this parameter study, the flaw shape is varied to investigate the influence of more narrow and elongated flaws. Table 8 displays the models used for this parameter study; the respective PDFs and CDFs are illustrated in Figure 23. Figure 24 presents the trends of the normalized AIC values, the mean values and the standard deviations.

Table 8 clearly demonstrates a minimum in the mean value, the standard deviation and the COV of the normal stress distribution when the flaw shape equals 0.8. This trend is extra visible in Figure 24b, where a plot is made of the mean values of normal stress at failure in function of the flaw shape. It results from Equations (6)–(9). These Equations calculate the corresponding geometry factor for the given flaw shape, and a maximum geometry factor of 0.734 is reached for a flaw shape equal to 0.833. Looking at Equation (2), this maximum leads to a higher stress intensity factor which means it will reach the fracture toughness sooner, resulting in a lower fracture load and, thus lower normal stresses at failure (i.e., a minimum for a flaw shape of 0.833).

The AIC calculation illustrates that there is no shift in the best fit for this parameter study. A normal distribution and a gamma distribution are good fits for all the models in this parameter study.

### 3.6. Maximum Flaw Depth

**Figure 25:**
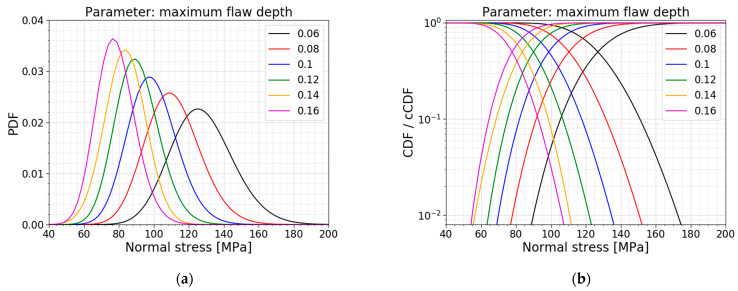
(**a**) PDF and (**b**) CDF for a varying maximum flaw depth.

**Figure 26:**
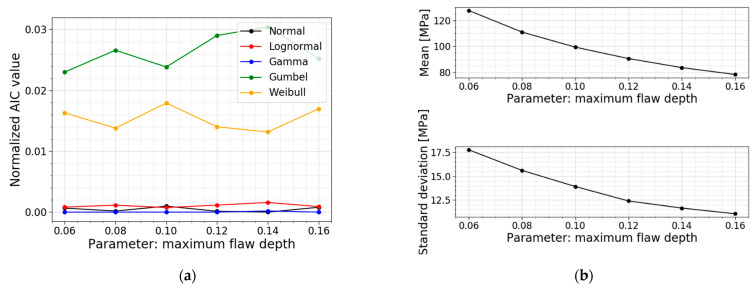
(**a**) AIC values and (**b**) the mean and st.dev. of the normal stress at failure for a varying maximum flaw depth.

The maximum flaw depth $a_{max}$ is the parameter used to simulate the depths of all the flaws that must be distributed over the glass surfaces. This parameter equals 0.1 mm, but the literature has already demonstrated a large spread for this value in reality. In the paper of Wereszczak et al. [22], different scanned glass surfaces showed the largest identified flaw on a glass pane going from 105 µm to 398 µm. This demonstrates the importance of performing a parameter study on this maximum flaw depth parameter. Table 9 displays the models used for this parameter study; all PDFs and all CDFs are visualized together in Figure 25. The trends of the normalized AIC values, the mean values and the standard deviations are presented in Figure 26. It can be concluded that the mean value and the standard deviation of the normal stress distribution decrease when the maximum flaw depth is increasing, and vice versa. The obtained COV is, however, approximately constant. Referring to Equation (2), a larger flaw depth results in a larger stress intensity factor which means it will reach the fracture toughness sooner, resulting in a lower fracture load and, thus lower normal stresses. 

The AIC calculation again shows no shift in the best fit for this parameter study. Both a normal distribution and a gamma distribution are again good fits for all the models in this parameter study.

### 3.7. Fracture Toughness

**Figure 27:**
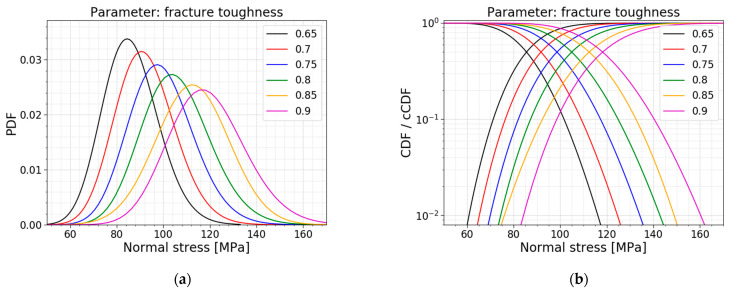
(**a**) PDF and (**b**) CDF for varying fracture toughness.

**Figure 28:**
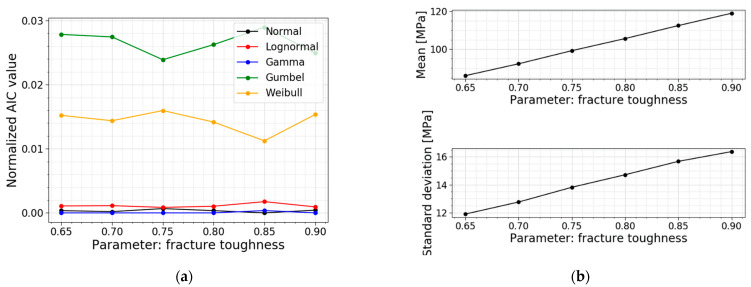
(**a**) AIC values and (**b**) the mean and st.dev. of the normal stress at failure for varying fracture toughness.

The last parameter study is the one on fracture toughness. The literature has proven that, at inert conditions, a fixed value of $0.75\;\mathrm{MPa}\sqrt{m}$ for the fracture toughness is a good assumption for soda–lime–silica glass [16,26,27]. Yet, different investigations have demonstrated that there is a certain variation in this parameter. Gong et al., (2001) [28] performed a statistical analysis of the fracture toughness of soda-lime-silica glass, determined by indentation. The experimental research displays a spread of the fracture toughness from 0.5 to 0.9 $\mathrm{MPa}\sqrt{m}$. [28]. The models used in this parameter study are based on the values of Gong et al., (2001). The results are displayed in Table 10, Figure 27 and Figure 28.

The results demonstrate an increase in the mean value and the standard deviation of the data of the normal stresses when increasing the fracture toughness. Note that the COV is more or less constant, resulting in a linear increase for both the mean value and the standard deviation. The increase in the fracture toughness allows for larger stress intensity factors before the glass breaks. This results in larger allowable normal stresses.

The best fits do not experience a shift for varying fracture toughness. The fracture toughness is a deterministic value and does not influence the best fit. Any change is the result of randomness in the Monte Carlo simulations. Again, both a normal distribution and a gamma distribution are good fits for all the models in this parameter study.

## 4. Conclusions

The strength prediction model by Osnes has been expanded with an AIC calculation to perform a model selection for every outcome of the SPM. This model selection proved that the gamma distribution was the best distribution fit for the data of the presented case study.

Based on a parameter study, it is concluded that the most appropriate model to represent the data of the normal stresses at failure was largely influenced by the following four parameters: the scale factor, the ratio of width to loading span, the ratio of loading span to support span, and the flaw density. As a general conclusion of this parametric study, the more flaws are subjected to the maximum tensile stresses, the more the Weibull distribution and the normal distribution become appropriate. Conversely, the less flaws are subjected to the maximum tensile stresses, the more the Gumbel distribution becomes the best fit. Overall, the gamma distribution performs well for all the simulated cases.

The parameter study further indicates that the mean value, standard deviation and COV of the glass panel strength increase while decreasing the scale factor, the ratio of the width to the loading span, the ratio of the loading span to the support span, or the flaw density. The mean value and the standard deviation of the data of the normal stresses increase more or less linearly, with a decrease in maximum flaw depth or an increase in fracture toughness. In these cases, the COV stays more or less constant. As a last conclusion, it was found that the mean value, the standard deviation and the COV of the data of the normal stresses reach a minimum when the scale factor of the flaw approaches a value of 0.833.

The performed parameter study and model selection gives a view of how the glass strength distribution changes while considering other parameter values. For example, the strength of a glass panel fresh from production (i.e., with a smaller flaw density) is more describable with a Gumbel distribution. In contrast, a glass panel already in service for several years (i.e., with a larger flaw density) corresponds more closely to a Weibull or normal distribution.

On the other hand, the performed parameter study of the flaw shape, the maximum flaw depth, and the fracture toughness highlight the importance of selecting the right values for the considered parameters. Until now, values have been chosen based on experimental results from the literature. However, this study emphasizes that a small difference in value can reasonably affect the strength distribution. 

In further research, the flaw shape, the maximum flaw depth, and the fracture toughness should be implemented as probabilistic values to account for the uncertainty of these parameters. Additionally, other cases (e.g., coaxial double-ring setups) could be checked to investigate whether the same trends can be found.

## Figures and Tables

**Table 2:** Mean value, standard deviation and COV for varying numbers of simulations.

Number of Simulations	Mean Value [MPa]	St.dev. [MPa]	COV [−]
50	101.43	15.14	0.149
100	96.84	13.11	0.135
500	98.84	13.97	0.141
1000	99.32	14.09	0.142
5000	98.96	13.84	0.140
10,000	98.86	13.86	0.140
50,000	98.91	13.88	0.140
100,000	98.93	13.83	0.140
500,000	98.96	13.84	0.140

**Table 3:** Simulations needed for convergence at a given $P_{q}$.

$P_{q}$	Number of Simulations
10^−1^	460
10^−2^	5 052
10^−3^	50 970
10^−4^	510 154
10^−5^	5 101 990

**Table 4:** Data for a varying scale factor.

Scale Factor [−]	Mean Value [MPa]	St.dev. [MPa]	COV [−]	Best Fit
0.25	148.10	42.11	0.284	Gumbel
0.5	117.62	21.94	0.187	Gamma
1 ^1^	99.28	13.71	0.138	Gamma
2	87.14	9.64	0.111	Normal
4	75.59	6.31	0.083	Normal

^1^ This scale factor is used in the Case Study of Section 2.2.3 and serves as a reference.

**Table 5:** Data for a varying ratio of the width to the loading span.

width/*L_l_* [−]	Mean Value [MPa]	St.dev. [MPa]	COV [−]	Best Fit
0.1	118.35	22.63	0.191	Lognormal
0.2	107.94	17.37	0.161	Gamma
0.3	103.07	15.46	0.150	Gamma
0.4	99.89	14.09	0.141	Gamma
0.43 ^1^	99.25	13.82	0.139	Gamma
0.5	97.85	13.31	0.136	Gamma
0.6	95.90	12.44	0.130	Normal
0.7	94.16	12.06	0.128	Gamma
0.8	93.06	11.61	0.125	Normal
0.9	92.21	11.25	0.122	Normal
1.0	91.26	11.03	0.121	Normal

^1^ This ratio of the width to the loading span is used in the Case Study of Section 2.2.3 and serves as a reference.

**Table 6:** Data for a varying ratio of the loading span to the support span.

*L_l_*/*L_s_* [−]	Mean Value [MPa]	St.dev. [MPa]	COV [−]	Best Fit
0.1	106.86	18.21	0.170	Gamma
0.2	104.37	16.61	0.159	Gamma
0.3	102.55	15.27	0.149	Gamma
0.4	100.55	14.28	0.142	Gamma
0.5 ^1^	99.25	13.82	0.139	Gamma
0.6	97.76	13.18	0.135	Normal
0.7	96.79	12.69	0.131	Normal
0.8	95.50	12.43	0.130	Gamma
0.9	94.95	12.09	0.127	Normal

^1^ This ratio of the loading span to the support span is used in the Case Study of Section 2.2.3 and serves as a reference.

**Table 7:** Data for a varying flaw density.

*ρ_flaw_* [flaws/cm^2^]	Mean Value [MPa]	St.dev. [MPa]	COV [−]	Best Fit
0.5	111.09	20.79	0.187	Lognormal
1.0	104.18	16.48	0.158	Gamma
1.5	101.29	14.61	0.144	Gamma
2.0 ^1^	99.25	13.82	0.139	Gamma
2.5	97.74	12.97	0.133	Gamma
3.0	96.89	12.52	0.129	Normal
3.5	95.73	12.10	0.126	Normal
4.0	94.96	11.72	0.123	Normal

^1^ This flaw density is used in the Case Study of Section 2.2.3 and serves as a reference.

**Table 8:** Data for a varying flaw shape.

*a/c* [−]	Mean Value [MPa]	St.dev. [MPa]	COV [−]	Best Fit
0.4	109.18	15.10	0.138	Gamma
0.6	100.40	14.16	0.141	Normal
0.8	98.20	13.48	0.137	Gamma
1.0 ^1^	98.83	13.94	0.141	Gamma
1.2	101.06	14.09	0.139	Gamma
1.4	104.39	14.44	0.138	Gamma
1.6	107.77	15.23	0.141	Normal

^1^ This flaw shape is used in the Case Study of Section 2.2.3 and serves as a reference.

**Table 9:** Data for a varying maximum flaw depth.

*a_max_* [mm]	Mean Value [MPa]	St.dev. [MPa]	COV [−]	Best Fit
0.06	127.72	17.78	0.139	Gamma
0.08	111.07	15.62	0.141	Gamma
0.10 ^1^	99.44	13.92	0.140	Gamma
0.12	90.62	12.40	0.137	Gamma
0.14	83.65	11.65	0.139	Normal
0.16	78.33	11.05	0.141	Gamma

^1^ This maximum flaw depth is used in the Case Study of Section 2.2.3 and serves as a reference.

**Table 10:** Data for varying fracture toughness.

*K_IC_* [MPa$\sqrt{m}$]	Mean Value [MPa]	St.dev. [MPa]	COV [−]	Best Fit
0.65	86.09	11.90	0.138	Gamma
0.70	92.35	12.76	0.138	Gamma
0.75 ^1^	99.25	13.82	0.139	Gamma
0.80	105.62	14.71	0.139	Gamma
0.85	112.46	15.67	0.139	Normal
0.90	118.96	16.37	0.138	Gamma

^1^ This fracture toughness is used in the Case Study of Section 2.2.3 and serves as a reference.

## Data Availability

Not applicable.
